# Altered Effective Connectivity among Core Neurocognitive Networks in Idiopathic Generalized Epilepsy: An fMRI Evidence

**DOI:** 10.3389/fnhum.2016.00447

**Published:** 2016-09-07

**Authors:** Huilin Wei, Jie An, Hui Shen, Ling-Li Zeng, Shijun Qiu, Dewen Hu

**Affiliations:** ^1^Department of Automatic Control, College of Mechatronics and Automation, National University of Defense TechnologyChangsha, China; ^2^Department of Medical Imaging, The First Affiliated Hospital of Guangzhou University of Chinese MedicineGuangzhou, China

**Keywords:** idiopathic generalized epilepsy, resting-state fMRI, effective connectivity, multivariate Granger causality, core neurocognitive networks

## Abstract

Idiopathic generalized epilepsy (IGE) patients with generalized tonic-clonic seizures (GTCS) suffer long-term cognitive impairments, and present a higher incidence of psychosocial and psychiatric disturbances than healthy people. It is possible that the cognitive dysfunctions and higher psychopathological risk in IGE-GTCS derive from disturbed causal relationship among core neurocognitive brain networks. To test this hypothesis, we examined the effective connectivity across the salience network (SN), default mode network (DMN), and central executive network (CEN) using resting-state functional magnetic resonance imaging (fMRI) data collected from 27 IGE-GTCS patients and 29 healthy controls. In the study, a combination framework of time domain and frequency domain multivariate Granger causality analysis was firstly proposed, and proved to be valid and accurate by simulation experiments. Using this method, we then observed significant differences in the effective connectivity graphs between the patient and control groups. Specifically, between-group statistical analysis revealed that relative to the healthy controls, the patients established significantly enhanced Granger causal influence from the dorsolateral prefrontal cortex to the dorsal anterior cingulate cortex, which is coherent both in the time and frequency domains analyses. Meanwhile, time domain analysis also revealed decreased Granger causal influence from the right fronto-insular cortex to the posterior cingulate cortex in the patients. These findings may provide new evidence for functional brain organization disruption underlying cognitive dysfunctions and psychopathological risk in IGE-GTCS.

## Introduction

Previous studies have revealed that patients with epilepsy suffer a higher incidence of psychosocial and psychiatric disturbances than healthy people (Mignone et al., 1970; Baker et al., 1996; Cutting et al., 2001; Gelisse et al., 2007). Idiopathic generalized epilepsy (IGE) is the most common type of epilepsy, which can be characterized by electroencephalography (EEG) recordings with generalized spike-and-waves or polyspike-waves (Engel, 2001; Hamandi et al., 2006). As one of the IGE subtypes, IGE patients with generalized tonic-clonic seizures (IGE-GTCS) suffer various neuropsychological impairments such as deficits in working memory, sustained attention, language, as well as executive functions (Hommet et al., 2006; Chowdhury et al., 2014). Prior studies have suggested that disruptions to these higher-order control processes may constitute a key aspect of psychopathology (Sridharan et al., 2008; Menon and Uddin, 2010). Therefore, distinguishing dysfunctional brain architecture may provide greater insight into the psychopathology in IGE-GTCS.

In recent years, identifying disturbed dynamic interactions of large-scale brain networks associated with cognitive and affective dysfunctions has shed new lights on the study of psychopathology. Of the many spatially distinct and functionally specialized stable brain networks, three have tended to be particularly crucial for understanding higher-order cognitive and perceptive processes thus described as core neurocognitive networks, they are: (1) the salience network (SN), involved in conflict monitoring, attention, as well as interoceptive and affective processes; (2) the default mode network (DMN), related to self-referential and social cognitive processes; and (3) the central executive network (CEN), associated with working memory, cognitive control implementation, and decision making in goal-directed behavior (Menon, 2011). Moreover, each of the three core neurocognitive networks are anchored in some key nodes that show strong intrinsic functional coupling as well as co-activation across different cognitively demanding tasks (Sridharan et al., 2008; Menon and Uddin, 2010). These key nodes are: (1) the right fronto-insular cortex (rFIC) and the dorsal anterior cingulate cortex (dACC) of the SN; (2) the ventromedial pre-frontal cortex (VMPFC) and the posterior cingulate cortex (PCC) of the DMN; as well as (3) the dorsolateral pre-frontal cortex (DLPFC) and the posterior parietal cortex (PPC) of the CEN. Particularly, the two key nodes of the SN have been highlighted in numerous researches, suggesting that the rFIC is crucial for initiating network switching between the CEN and the DMN (Sridharan et al., 2008; Menon and Uddin, 2010; Uddin et al., 2011), and the dACC most closely associated with conflict monitoring to mediate higher-order cognitive processes (Botvinick et al., 2004; Menon, 2011). Investigating disruptions to functional dynamics among these key nodes is beginning to identify an important aspect of dysfunctions in psychopathology, thus the three core neurocognitive networks represented by the associated key nodes have been concluded as a “triple network” model (Menon, 2011). Aberrant interconnectivity and intrinsic organization of the triple network is characteristic of various neurological and psychiatric disorders, such as schizophrenia, depression, anxiety disorders and autism (Paulus and Stein, 2006; Walter et al., 2009; White et al., 2010; Uddin et al., 2015), and is likely to provide better understanding of fundamental brain mechanisms underlying cognitive dysfunctions and psychopathology in IGE-GTCS.

Measurement of causal influence that a system exerts over one other is called effective connectivity (Friston et al., 1993). Applying effective connectivity to brain network analysis can obtain full understanding of the network interaction structure including the strength and direction of information flow between brain regions. Granger causality analysis, as an important analytical technique of effective connectivity, has been widely applied in cognitive neuroscience studies since it can measure directional dependence between time courses without any prior model specifications. In Granger causality definition, time course *X*_2_ causes time course *X*_1_ if combined past value of both *X*_1_ and *X*_2_ can significantly improve the prediction accuracy of current value of *X*_1_, rather than using the past value of *X*_1_ alone (Granger, 1969; Seth, 2010). Granger causality is often estimated with multivariate autoregressive (MVAR) modeling of the time courses, and has various time domain as well as frequency domain formulations, including conditional Granger causality (Geweke, 1984), partial Granger causality (Guo et al., 2008), directed transfer function (DTF) (Kaminski et al., 2001), and partial directed coherence (PDC) (Baccalá and Sameshima, 2001), etc. The mentioned time domain Granger causality measures are the straightforward generalization of the notion of Granger causality thus easy to comprehend, the introduced frequency domain Granger causality measures could describe the dynamics of causal relationships between time courses by evaluating Granger causality over different frequency portions (Sato et al., 2009). Based on these, the combined performance of Granger causality analysis in two domains is expected to provide more accurate and informative analysis results.

Little is known about the alteration of effective connectivity among core neurocognitive networks underlying cognitive impairments and psychopathology in IGE-GTCS. Additionally, to our knowledge, no IGE study has conducted multivariate Granger causality analysis in both time and frequency domains and presented the combined analysis results. In the current study, we examined IGE-related changes in effective connectivity across core neurocognitive brain networks using resting-state functional magnetic resonance imaging (fMRI), combining time domain and frequency domain multivariate Granger causality analysis. We hypothesized that the altered causal interactions likely occur among the key nodes of the SN, DMN, and CEN in IGE-GTCS, which may underline cognitive dysfunctions, improving our understanding of the psychopathological mechanism of IGE-GTCS.

## Materials and methods

### Subjects

Twenty-seven right-handed IGE-GTCS patients (age 24.93 ± 5.95 years; education 10.59 ± 2.58 years; eight female; epilepsy duration 7.76 ± 5.62 years; age of onset 17.13 ± 6.11 years) were recruited in the study. The diagnosis was determined by a comprehensive evaluation including detailed history, video-EEG telemetry, and neuroimaging. All patients had IGE with GTCS only according to the International League against Epilepsy (ILAE) classification, and met the following inclusion criteria: (i) presence of typical clinical symptoms of GTCS, including myoclonus, loss of consciousness, and no partial seizures; (ii) presence of generalized spike-and-wave or polyspike-wave discharges in their scalp EEG; (iii) no focal abnormality in routine structural MRI examinations; and (iv) no obvious history of etiology. All patients were treated with antiepileptic drugs (AEDs), but received no medication for at least 48 h prior to the MRI scanning. All patients had been seizure-free for at least 1 month prior to the MRI scanning.

Twenty-nine right-handed healthy subjects (age 26.93 ± 7.54 years; education 11.45 ± 2.40 years; 12 female) were recruited, with gender, age, and education level demographically matched. All participants had no mass lesion (including tumor, vascular malformation or malformations of cortical development), traumatic brain injury or history of neurological or psychiatric disorder. This study was approved by the Ethics Committee of Guangdong 999 Brain hospital, and all participants provided written informed consent.

### MRI data acquisition and pre-processing

For the resting-state fMRI scan acquired at a 1.5-T Philips Intera MR scanner, all subjects were instructed to stay awake, keep their eyes open, and minimize head movement; no other task instruction was provided. For the patients, scans were conducted during interictal without combined EEG confirmation. All fMRI images were collected using a gradient-echo echo-planar pulse sequence sensitive to blood-oxygenation-level-dependent (BOLD) contrast with the following parameters: TR/TE = 3000/30 ms, thickness/gap = 4.5/0 mm, field of view (FOV) = 230 × 230 mm, flip angle (FA) = 90°, matrix = 128 × 128, and slices = 31. Each resting-state fMRI run lasted 8 min, obtaining 160 volumes.

For each subject, the first five volumes of the scanned data were discarded to allow for T1-equilibration effects, and then the fMRI data were pre-processed with SPM8 package (Welcome Department of Cognitive Neurology, Institute of Neurology, London, UK, http://www.fil.ion.ucl.ac.uk/spm), included the following steps (Zeng et al., 2012): (1) slice timing correction; (2) rigid body correction for head motion; (3) atlas registration with an EPI template in the Montreal Neurological Institute (MNI) atlas space, resampling to 3-mm isotropic voxels; (4) spatially smoothing using an 8-mm full-width half-maximum (FWHM) Gaussian kernel; and (5) regressing out nine nuisance signals including signals averaged from white matter, cerebrospinal fluid, and the whole brain, and six parameters obtained by head motion correction. Temporal filtering was not conducted as with some prior studies (Hamandi et al., 2006; Wu et al., 2013), so that the whole effective frequency band of the fMRI data could be included in the frequency domain Granger causality analysis (see Section Effective connectivity: time and frequency domains multivariate Granger causality measures below). After calculation, no subjects were removed due to excessive motion (translation > 2 mm and rotation >2°); there was no significant difference in mean motion between the two groups (*p* = 0.32, two-tailed two-sample *t*-test; Zeng et al., 2014), thus the effective connectivity would be less probably affected by the head motion (Van Dijk et al., 2012).

### Region of interest definition and time course extraction

Six functional regions of interest (ROIs) were selected, including the rFIC and the dACC of the SN, the VMPFC and the PCC of the DMN, as well as the rDLPFC and the rPPC of the CEN. The coordinates of the ROIs (Table 1) were set according to a published study delimiting these regions in an independent dataset (Uddin et al., 2011). In that study, MNI coordinates of peak voxels (voxels with the highest z-scores) of the six regions chosen from ICA maps were defined as the centers of the ROIs. In our study, the final ROIs were defined as 8 mm radius spheres centered on the coordinates, and the mean time course in each ROI was extracted by averaging the time courses of all voxels within the ROI. At last, each mean time course of the ROIs was detrended and its temporal mean was removed for further analysis. All the time courses were covariance stationarity (i.e., unchanging mean and variance) after time course pre-processing.

**Table 1:** **Coordinates of ROIs**.

**Region**	**BA**	**Peak MNI coordinates (mm)**
**SALIENCE NETWORK (SN)**		
Right fronto-insular cortex (rFIC)	47	39, 23, −4
Dorsal anterior cingulate cortex (dACC)	24	6, 24, 32
**DEFAULT MODE NETWORK (DMN)**		
Ventromedial pre-frontal cortex (VMPFC)	11	−2, 38, −12
Posterior cingulate cortex (PCC)	23/30	−6, −44, 34
**CENTRAL EXECUTIVE NETWORK (CEN)**		
Right dorsolateral pre-frontal cortex (rDLPFC)	9	46, 20, 44
Right posterior parietal cortex (rPPC)	40	52, −52, 50

### Effective connectivity: time and frequency domains multivariate granger causality measures

On the basis of MVAR modeling, we intended to calculate the effective connectivity strength in both time and frequency domains, thus the well-chosen Granger causality measures in two domains were introduced in the current study, they are: partial Granger causality (Guo et al., 2008) in time domain analysis, and PDC (Baccalá and Sameshima, 2001) in frequency domain analysis. The formalism for these Granger causality measures is given in Appendix.

To obtain the time domain and frequency domain Granger causality measures between each pair of the six ROIs for each subject, the following steps were conducted: (1) MVAR model estimation: six time courses were fit to obtain the unrestricted autoregressive model (see Appendix for details), the model order was set to 1 determined by Bayesian information criterion (BIC), and the regression coefficients were estimated using standard least squares optimization. (2) Calculation of time domain Granger causality measures: for each pair of the six time courses in both directions, the partial Granger causality and the DOI were calculated (see Equations A11 and A12 in Appendix); thus, 30 (6 × 5) individual partial Granger causality values of time domain Granger causal links and 30 DOI terms of time domain Granger causal links were obtained for each subject. (3) Calculation of frequency domain Granger causality measure: for each pair of the six time courses in both directions, the PDC was calculated (see Equation A13 in Appendix) every 0.001 Hz of the interesting frequency range [0, Fs/2], where Fs is the sampling rate of the fMRI data (i.e., 1/TR); thus 30 × 168 individual PDC values of frequency domain Granger causal links were obtained for each subject.

### Constructing within-group effective connectivity graph

**Figure 1:**
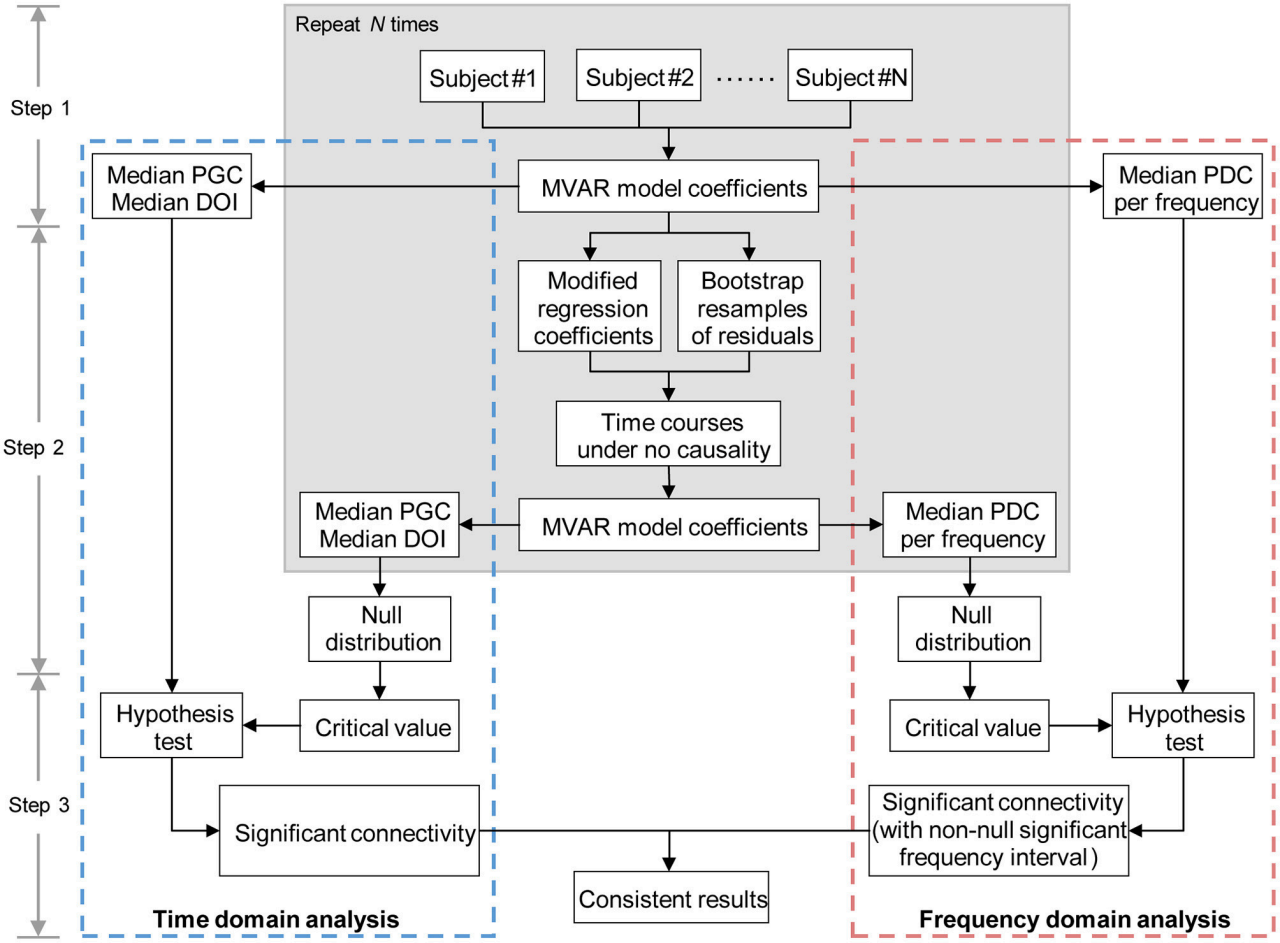
**Diagram representing the main steps of combination method of time and frequency domains multivariate Granger causality analyses**. MVAR, multivariate regressive; PGC, partial Granger causality; DOI, difference of influence; PDC, partial directed coherence.

Having computed the Granger causality measures in both time and frequency domains, we proceeded to construct effective connectivity graph for each group, respectively. Since all the Granger causality measures used in the study lack known statistical distributions (Seth, 2010), the creation of empirical null distributions that hypothesize no causality between ROIs is of great importance. Meanwhile, we hypothesized that the combined performance of time domain and frequency domain analysis of multivariate Granger causality would present more accurate and informative analysis results. Therefore, based on the procedure conducted in Sato et al. (2009) and Havlicek et al. (2010), we proposed a combination framework of time domain and frequency domain multivariate Granger causality analysis to evaluate the direct causal interactions between time courses. An overview of this method (see Figure 1) is given below, and each step is described in detail as follows:

Step 1 Fit MVAR model for the time courses of each subject separately to obtain the model coefficients (including regression coefficients and residuals, see Appendix for details), then calculate the time and frequency domains Granger causality measures for each subject (see Equations A11–A13 in Appendix). Record the median values of each Granger causality measure across subjects.
Step 2 For each subject, resample the residuals (bootstrap resampling for *N* repetitions) and set the regression coefficients *A*_*ij*_(*l*), *l* = 1, …, *p* to zero (see Appendix for details) when assessing the Granger causality from time courses *j* to *i*, the other coefficients remain as originally estimated in step 1. Then simulate a multivariate time courses based on the modified MVAR model coefficients to generate time courses under the null hypothesis of “no Granger causality” from time courses *j* to *i*. After that, calculate the Granger causality measures of the simulated time courses (see Equations A11–A13 in Appendix), then record the median values of the Granger causality measures across the simulated samples. Repeat this step until the desired number of repetition (*N* times) is achieved. When finished, the null distributions of the median Granger causality measures are obtained. Note, in general, the value of *N* = 200–5000 is sufficient (in the current study, we set *N* = 1000) (Efron and Tibshirani, 1994).
Step 3 Estimate the critical value (defined as the (1−α) quantile, α = 0.05, FDR corrected; Seth, 2010) of each null distribution, and take the critical value as significance threshold. For time domain analysis, a per-interaction significance threshold is obtained above which the median values of the Granger causality measures recorded in step 1 are assumed to be significant. For frequency domain analysis, we get a per-interaction-per-frequency significance threshold; the significant effective connectivity is thus defined as the connection which has non-null significant frequency interval. Finally, the consistent results of time domain and frequency domain analysis are determined as significant effective connectivity given by the proposed method.

The validity and improvement in resulting accuracy of the proposed method is proved by several toy models in the following subsection (see Section Simulations). For the IGE study, we used the median partial Granger causality and median PDC to determine the significant connectivity in time domain and frequency domain analysis, respectively. Finally, the significant effective connectivity was defined as the connection that was significant in both time domain and frequency domain analysis, and the within-group effective connectivity graph was thus composed of the significant effective connections of each group. In addition, the significant connections identified by DOI terms in time domain analysis were also recorded as a subset of the final results.

### Evaluating between-group effective connectivity difference

Among the connections that exhibited significant Granger causality in at least one group (obtained in Section Constructing within-group effective connectivity graph), we further assessed the presence of significant group differences in both time domain and frequency domain Granger causality definition. In time domain analysis, Mann-Whitney *U*-tests (*p* < 0.05, FDR corrected) were applied across the 30 time domain Granger causal links to assess the presence of significant group differences (Sridharan et al., 2008). In frequency domain analysis, for each link, Mann-Whitney *U*-tests (*p* < 0.05, FDR corrected) were applied across the 168 frequency slices to determine the group-level significant frequency interval of that link. And finally the links with non-null significant frequency intervals were taken as the interesting results in frequency domain analysis.

## Results

### Simulations

Two typical and widely used toy models (Baccalá and Sameshima, 2001; Seth, 2010) were presented here to demonstrate the validity and improvement in resulting accuracy of the proposed combination framework described in Section Constructing within-group effective connectivity graph. In the simulation experiments, the same methods of time course pre-processing (including detrend and removal of temporal mean), MVAR model estimation (using standard least squares optimization to calculate the regression coefficients and residuals, and setting the model order as the real model order of each toy model), time and frequency domains Granger causality calculation, and significance testing (1000 times repetition to get the significance thresholds) that described in Section Effective connectivity: time and frequency domains multivariate Granger causality measures and Constructing within-group effective connectivity graph were conducted to the toy models.

**Model 1**. Suppose that four simultaneously observed time courses were generated by the equations:

(1) $$\begin{array}{l} \begin{array}{l} x_{1}\left(n\right)=0.95\sqrt{2}x_{1}\left(n-1\right)-0.9025x_{1}\left(n-2\right)+\omega_{1}\left(n\right)\\ x_{2}\left(n\right)=0.5x_{1}\left(n-2\right)+\omega_{2}\left(n\right)\\ x_{3}\left(n\right)=-0.4x_{4}\left(n-3\right)+\omega_{3}\left(n\right)\\ x_{4}\left(n\right)=0.35x_{4}\left(n-2\right)+\omega_{4}\left(n\right) \end{array} \end{array}$$

**Figure 2:**
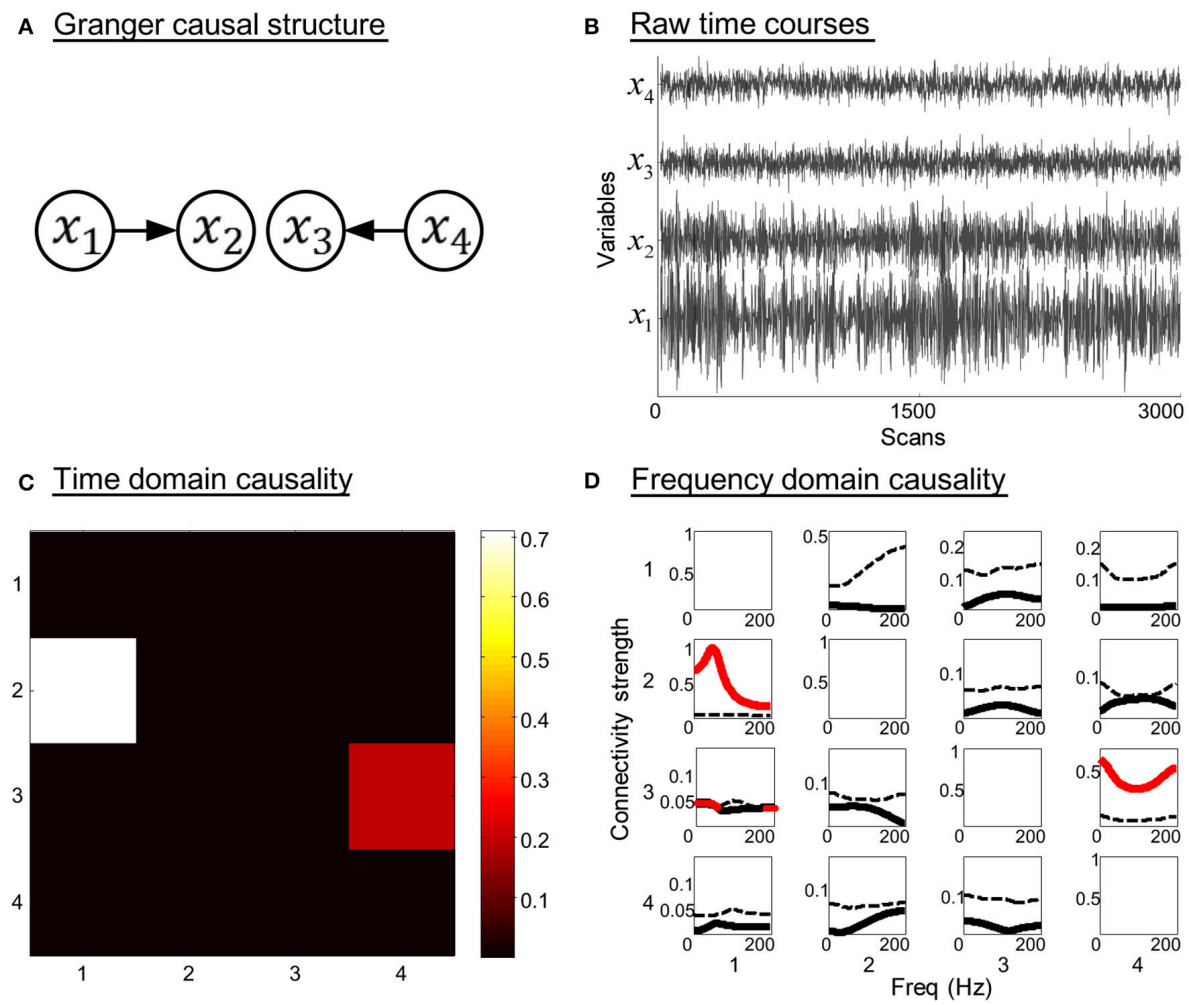
**Simulation results of toy model 1**. **(A)** Granger causal structure of the variables. **(B)** Raw time courses of the variables. **(C)** The colormap of partial Granger causality values in time domain analysis. **(D)** The spectrum of significance thresholds (black dotted line) and partial directed coherence (PDC) values (black solid line, values greater than the thresholds are highlighted in red) in frequency domain analysis. Note: in **(C,D)** the direction of causality is from column to row.

The model contains two direct Granger causal influences, i.e., connections from *x*_1_ to *x*_2_, and from *x*_4_ to *x*_3_. The model order is three, ω_1_ ~ ω_4_ are zero-mean uncorrelated white processes with identical variances. The signal to noise ratio (SNR) of the generated time courses is 0.01. Figure 2 illustrates the simulation results. The Granger causal structure and the raw time courses of each variable are shown in Figures 2A,B. The time domain Granger causality analysis result is expressed as a colormap in Figure 2C. As expected, the partial Granger causality values of the connections from *x*_1_ to *x*_2_, and from *x*_4_ to *x*_3_ were significantly larger and exceeded the corresponding thresholds. Figure 2D shows the PDC values (black solid line) and significance thresholds (black dotted line) of each connection. The significant frequency intervals were highlighted in red. Using the PDC representation we could observe the dynamics of causal relationships between time courses. It can be seen that, except for two correct causal influences, the connection from *x*_1_ to *x*_3_ was misjudged in frequency domain analysis. Obviously, when we conducted the proposed combination method, only the corrected causal interactions would be identified.

**Model 2**. A more complicated system that contains indirect causal influence was generated by the equations:

(2) $$\begin{array}{lll} x_{1}(n) & = & 0.95\sqrt{2}x_{1}(n-1)-0.9025x_{1}(n-2)+\omega_{1}(n)\\ x_{2}(n) & = & 0.5x_{1}(n-2)+\omega_{2}(n)\\ x_{3}(n) & = & -0.4x_{1}(n-3)+\omega_{3}(n)\\ x_{4}(n) & = & -0.5x_{1}(n-2)+0.25\sqrt{2}x_{4}(n-1)\\ & & +\;0.25\sqrt{2}x_{5}(n-1)+\omega_{4}(n)\\ x_{5}(n) & = & -0.25\sqrt{2}x_{4}(n-1)+0.25\sqrt{2}x_{5}(n-1)+\omega_{5}(n) \end{array}$$

**Figure 3:**
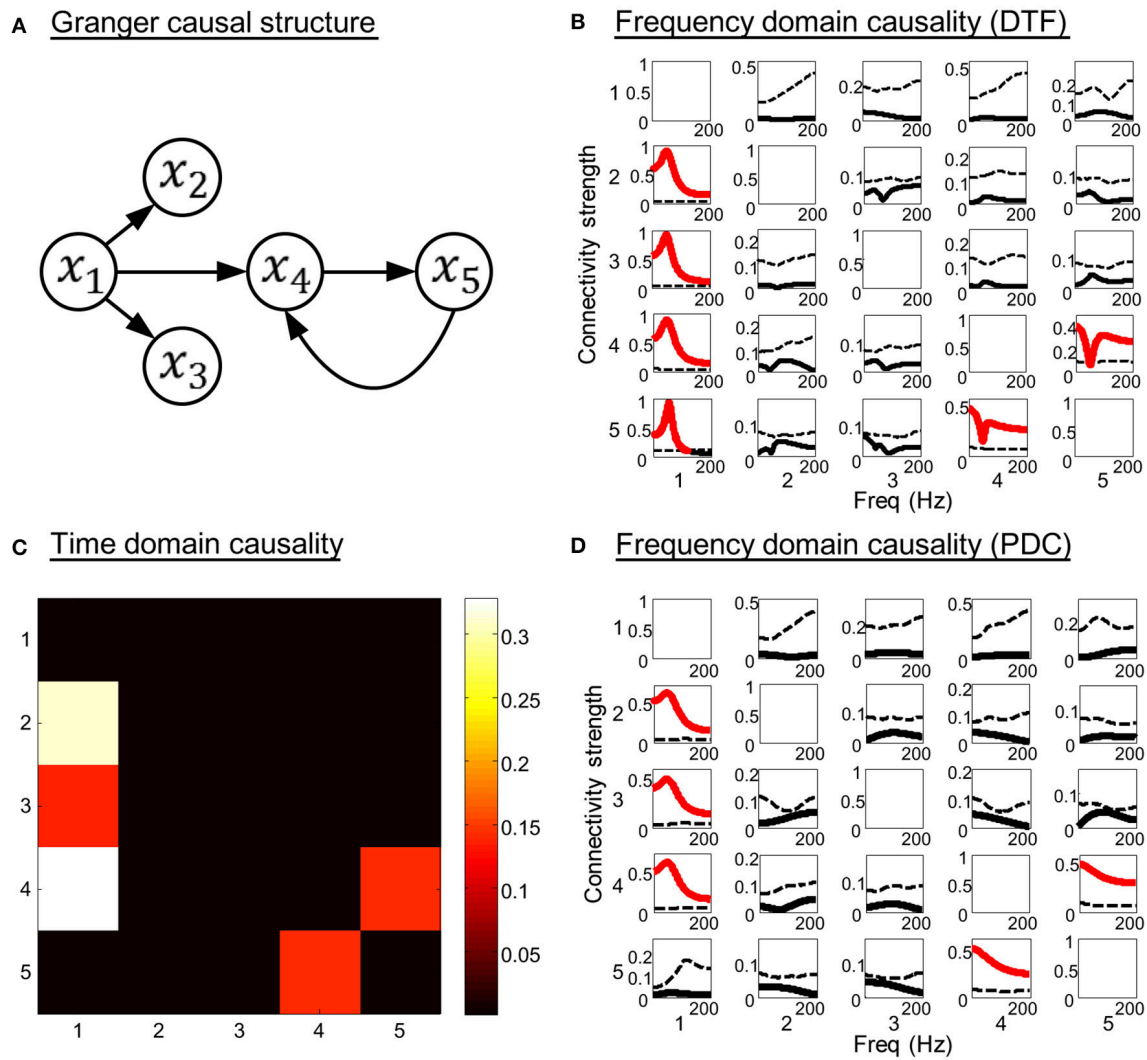
**Simulation results of toy model 2**. **(A)** Granger causal structure of the variables. **(B)** The spectrum of significance thresholds (black dotted line) and directed transfer function (DTF) values (black solid line, values greater than the thresholds are highlighted in red). **(C)** The colormap of partial Granger causality values in time domain analysis. **(D)** The spectrum of significance thresholds (black dotted line) and partial directed coherence (PDC) values (black solid line, values greater than the thresholds are highlighted in red) in frequency domain analysis. Note: in **(B–D)** the direction of causality is from column to row.

In this three order system, *x*_1_ is a direct source to *x*_2_, *x*_3_, and *x*_4_, bidirectional connectivity exists between *x*_4_ and *x*_5_. There is no direct coupling from *x*_1_ to *x*_5_. The SNR of the generated time courses is 0.01. The simulation results are shown in Figure 3. Figures 3C,D illustrate the time domain and frequency domain analysis results, respectively. In addition, the results given by DTF (Kaminski et al., 2001) are presented in Figure 3B as a reference (see Equation A14 in Appendix). It is obvious that both the time domain partial Granger causality and frequency domain PDC could correctly detect all the direct causal influences, while the DTF mistakenly identified the indirect causal influence from *x*_1_ to *x*_5_. These results indicate that the Granger causality measures we used in the study could avoid the influence of indirect causal relationship.

Based on the above analysis, we can conclude that the analytical methods and Granger causality measures adopted in the study can efficiently detect the direct causal relationships between time courses, and the combined approach takes the consistent results of the two domains' analyses, which can be seen as a double verification process to present more accurate and confident results. The simulation results were stable under different noise condition. Therefore, using the proposed method in Section Constructing within-group effective connectivity graph is considered to present a convincing result for fMRI data analysis.

### Within-group effective connectivity graph

**Figure 4:**
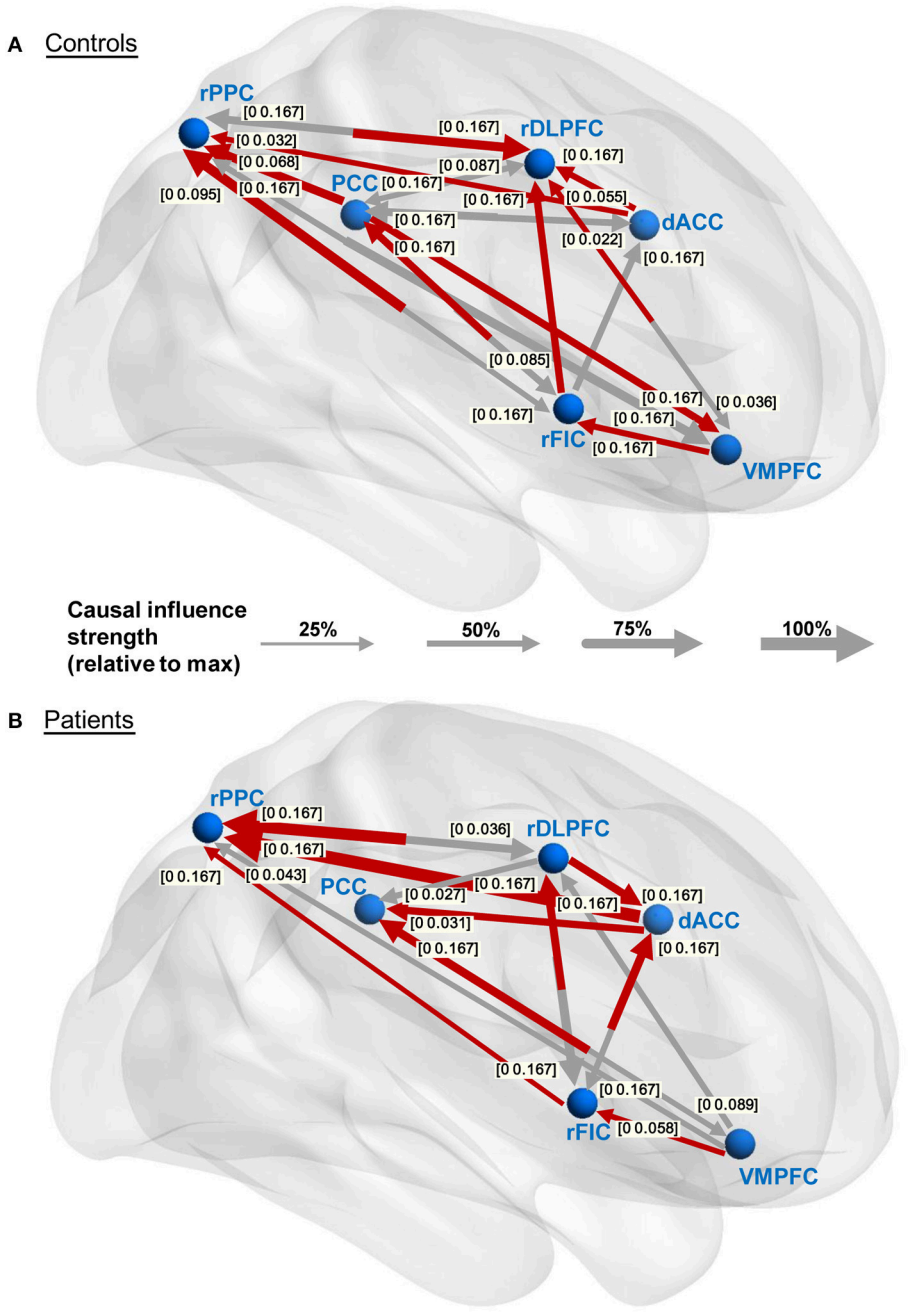
**Within-group effective connectivity graphs**. **(A,B)** Effective connectivity graphs of the healthy controls and IGE-GTCS patients, respectively. The connecting lines are weighted according to the normalized partial Granger causality values. The numbers next to the arrowheads indicate the significant frequency intervals of the corresponding connections. The significant connections showing a dominant direction of influence (significant DOI term) are highlighted in red.

The causal connectivity graphs of healthy controls and IGE-GTCS patients are presented in Figures 4A,B. The connecting arrows are weighted according to the strengths of the time domain causal influences (partial Granger causality values normalized by the maximum partial Granger causality value). Meanwhile, each significant connection is respectively marked with the frequency interval where the PDC values are higher than the significance thresholds. And finally a subset of the significant connections that showed a dominant direction of influence (significant DOI term) are highlighted in red in the same figure. It was observed that comparing to the healthy controls (21 influences), the IGE-GTCS patients (16 influences) established less causal connections among the six ROIs.

### Between-group effective connectivity differences

**Figure 5:**
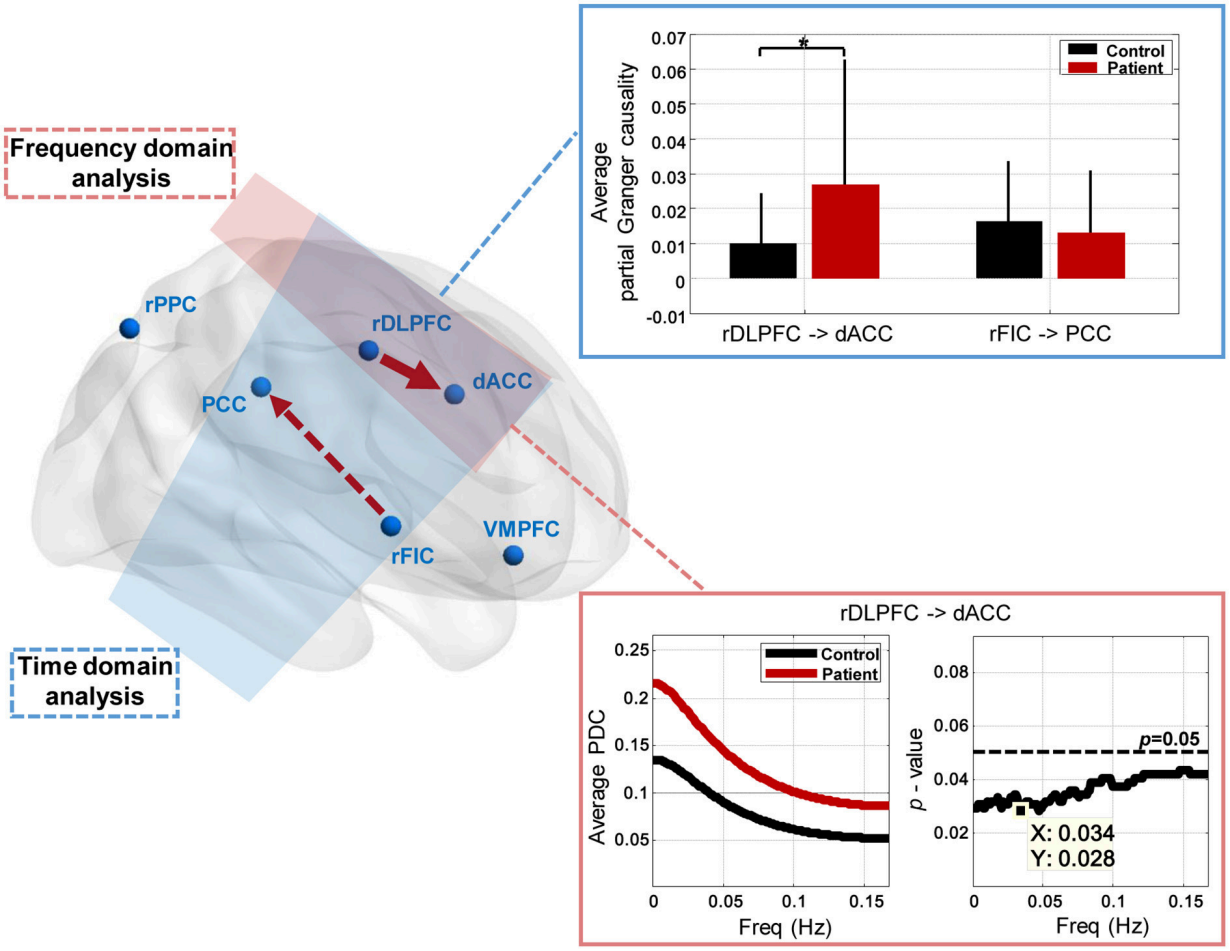
**Between-group effective connectivity differences**. Two connections exhibit significant between-group difference revealed by Mann-Whitney *U*-tests in time domain analysis, including the increased connection from the rDLPFC to the dACC (^*^p < 0.05, FDR corrected), and the decreased connection from the rFIC to the PCC (*p* < 0.05, uncorrected) in patients than healthy controls. The connections' means and standard errors of partial Granger causality values across subjects within each group are illustrated in the blue box. Frequency domain analysis also reveals the enhanced connection from the rDLPFC to the dACC (*p* < 0.05, FDR corrected) in patients. The mean partial directed coherence (PDC) values across subjects within each group, and the *p*-value spectrum are shown in the pink box.

The group differences of effective connectivity are illustrated in Figure 5. In time domain analysis, two connections exhibited significance, i.e., the increased causal influence from the rDLPFC to the dACC (*p* < 0.05, FDR corrected), and the decreased causal influence from the rFIC to the PCC (*p* < 0.05, uncorrected) in the IGE-GTCS patients relative to healthy controls. The connections' means and standard errors of partial Granger causality values across subjects within each group were illustrated in the blue box in Figure 5. Meanwhile, frequency domain analysis also found the enhanced causal influence from the rDLPFC to the dACC (*p* < 0.05, FDR corrected) in patients than healthy controls. The mean PDC values across subjects within each group, as well as the *p*-value spectrum of this significant connection were shown in the pink box. It can be seen that the group difference of this causal influence was significant in a band of frequencies, [0 0.167] Hz, and the minimum *p*-value (*p* = 0.028) was obtained at 0.034 Hz.

## Discussion

Human high-level attention and cognitive control processes rely on the well-balanced dynamic interactions between large-scale brain networks, and three core neurocognitive networks including the SN, DMN, and CEN have been highlighted in the study of psychopathology. Our prior work used static as well as dynamic measures of functional connectivity, however, did not evaluate effective connectivity among brain networks for cognitive dysfunctions and psychopathological risk in IGE-GTCS (Wei et al., 2015). In this study, we have proposed a combination framework of time domain and frequency domain multivariate Granger causality analysis, to reveal alterations in direct causal relationship across key nodes of the SN, DMN, and CEN in the IGE-GTCS patients relative to the healthy controls. The key findings of the study include: (1) the establishment of less causal interactions among the key nodes in the patients compared with healthy controls; (2) two SN-involved effective connectivity that exhibited significant group difference, they are: enhanced causal influence from the rDLPFC to the dACC (*p* < 0.05, FDR corrected) throughout the whole evaluated frequency range ([0 0.167] Hz) in patients than healthy controls revealed by both the time and frequency domains analyses, and decreased causal influence from the rFIC to the PCC (*p* < 0.05, uncorrected) in patients than healthy controls given by the time domain analysis. These findings provide new insights into the brain functional architecture of IGE-GTCS.

### Methodological considerations

Several methodological considerations in the present study need to be addressed aforehand. First, the basis of multivariate Granger causality and well-chosen time and frequency domains Granger causality measures ensure the indirect causality between ROIs to be eliminated, and this could be certified by the simulation results of the toy model 2 in Section Simulations. Second, the MVAR model order was set to 1 for all subjects according to the BIC criterion, thus we evaluated the Granger causal relationship between ROIs with a maximum time delay of 3 s (since TR is 3 s). The low model order is common and recommended in several Granger causality studies using resting-state fMRI data considering the low time resolution of fMRI data itself (Sato et al., 2010; Hamilton et al., 2011). Third, to our knowledge, no previous study has evaluated the Granger causal connectivity in IGE-GTCS combined the time domain and frequency domain analysis. In the current study, we have revealed aberrant causal interactions among the core neurocognitive networks in IGE-GTCS confirmed by analyses in two domains. Besides, a combination framework of time domain and frequency domain multivariate Granger causality analysis was proposed, and the improvement of accuracy using this method was verified by the simulation experiments. This general combination framework can also be used in other multisubject studies when effective connectivity measured by multivariate Granger causality is needed.

### The causal relationship between the rFIC and the PCC

In the current study, the controls group established bidirectional effective connectivity between the rFIC and the PCC (the influence from the rFIC to the PCC also exhibited significant DOI value), while none of these two connections was significant in the patients group (see Figure 4). Further, the between-group analysis based on time domain partial Granger causality revealed that the effective connectivity from the rFIC to the PCC exhibited significance (*p* < 0.05, uncorrected, Mann-Whitney *U*-test), with decreased connectivity strength in the patients relative to the healthy controls (see Figure 5). It is well known that the function of the SN is to identify internal and extra-personal stimuli to guide flexible behavior (Corbetta and Shulman, 2002; Seeley et al., 2007), and the DMN is associated with spontaneous activities and internally oriented cognition (Raichle et al., 2001). Previous task-based as well as resting-state fMRI studies using Granger causality analysis have confirmed that there exists effective connectivity between the SN and the DMN (Sridharan et al., 2008; Uddin et al., 2011). Among these researches, one commonly approved conclusion is that the rFIC acts as a critical causal outflow hub in initiating control signals to activate the CEN and deactivate the DMN, thus provides an interpretation of the directionality of signaling from the rFIC to the PCC. Moreover, a relevant neurodevelopmental study reported that the Granger causal influence from the rFIC to the PCC was significant in the adults group while vanished in the children, suggesting the maturation of rFIC-related causal connectivity is crucial for the sophisticated cognitive abilities (Uddin et al., 2011). For the causal influence from the PCC to the rFIC, Uddin et al. (2009) using Granger causality analysis provided evidence that the PCC may negatively regulate activity in the SN. Such an information inflow may be interpreted as a feedback circuit establishment that suppresses the activity of the DMN in a primed state to make better preparation for the rFIC to release cognitive control processes when salient stimuli occur.

For the frequency-domain interpretation of the causal interactions between the rFIC and the PCC, the significant frequency intervals of the two connections in the control group showed that the PCC conducted causal influence on the rFIC for the lower frequencies ([0 0.085] Hz). This is reasonable given the fact that the PCC as a key node of the DMN, is responsible for information integration in the spontaneous low-frequency range (Leech and Sharp, 2014). Meanwhile, the drive from the rFIC to the PCC was significant throughout the evaluated frequency interval, [0 0.167] Hz, probably indicating that the brain responses for cognitive control processes in switching between exogenous and endogenous stimuli are needed for the whole spectrum of signal changing frequencies (see Figure 4A).

Based on the above, we inferred that the bidirectional effective connectivity between the rFIC and the PCC may be associated with well-balanced performance in cognitive flexibility, with which one can flexibly switch between mental processes to appropriately react to salient events in the environment (Scott, 1962). Additionally, prior study has suggested that the active dynamic interactions among brain networks are indispensable for adaptive and flexible cognition and behavior (Cole et al., 2013), while the IGE-GTCS patients (16 influences) established less causal connections among the SN, DMN, and CEN relative to the healthy controls (21 influences, see Figure 4). For all the aforementioned proofs, we inferred that the hypoconnectivity of the patients group, especially the decreased causal influence from the rFIC to the PCC, may be associated with impaired cognitive abilities as well as mental inflexibility in IGE-GTCS (Hommet et al., 2006; Chowdhury et al., 2014).

### The causal relationship between the dACC and the rDLPFC

Both the time domain and frequency domain analysis in our study consistently revealed the significantly enhanced effective connectivity (*p* < 0.05, FDR corrected, Mann-Whitney *U*-test) from the rDLPFC to the dACC in the patients relative to the healthy controls (see Figure 5). Interestingly, the within-group connectivity graphs indicated that the direction of the Granger causality between the rDLPFC and the dACC is opposite in the two groups, i.e., the dACC drives the rDLPFC (also with significant DOI value) in the controls while the rDLPFC drives the dACC (also with significant DOI value) in the IGE patients (see Figure 4). Since both the ACC and the DLPFC are co-activated in cognitive control processing and tests of sustained attention (Adler et al., 2001; Miller and Cohen, 2001), the dissociation and functional interactions of the two areas have arouse the interests of the researchers (Kondo et al., 2004; Dosenbach et al., 2007; Seeley et al., 2007). In an event-related fMRI study, Macdonald et al. (2000) conducted a task-switching vision of the Stroop task and suggested that the DLPFC (Brodmann's area (BA) 9) supports implementation of control, while the ACC (BA 24 and BA 32) is responsible for performance monitoring. Furthermore, based on the conflict hypothesis of the ACC, Kerns et al. (2004) explored whether ACC activity associated with conflict and error trial predicted pre-frontal cortex activity under Stroop task, and concluded that once the ACC detects conflicts, it modulates the strength of the rDLPFC (BA 9 and BA 8) representations, which then executes appropriate cognitive control and products corresponding behavioral adjustments. Our study revealed the effective connectivity from the dACC (BA 24) to the rDLPFC (BA 9) in the healthy controls, which may underline the existence of neural circuitry in terms of resting-state Granger causality supporting the above cognitive control processes. By contrast, the establishment of the significantly enhanced causal influence from rDLPFC to dACC in the patients may thus indicate a disruption to the well-organized cognitive control processes, and probably associated with cognitive dysfunctions in IGE-GTCS, such as deficits in working memory, sustained attention, as well as executive dysfunction (Mirsky et al., 2002). This altered causal influence as well as the aberrant connection from the rFIC to the PCC demonstrate that the IGE-GTCS patients exhibit inappropriate mapping with the SN. The findings together with various prior studies highlight the critical role of SN in connecting with DMN and CEN (Sridharan et al., 2008; Menon, 2011; Uddin et al., 2011), which provide informative evidence for the understanding of the cognitive dysfunctions and psychopathological mechanism of IGE-GTCS.

In addition, prior study using Granger causality analysis on EEG/fMRI data of IGE patients found the frontal lobe had the maximum net causal strength, suggesting that frontal and parietal areas were the initiation of absence seizures (Szaflarski et al., 2010). Similarly, our study using both time domain and frequency domain multivariate Granger causal analysis revealed the significantly enhanced causal influence directed from the rDLPFC to the dACC throughout the whole evaluated frequency range ([0 0.167] Hz) in the IGE-GTCS patients, which may as well indicate that the pre-frontal cortex is probably the initiation of GTCS.

Evaluating hemodynamic response function (HRF) effects in the Granger causality analysis of BOLD-fMRI data is a controversial topic (Barnett and Seth, 2014). Noticing that BOLD-fMRI is an indirect transformation of underlying neural activity and Granger causality is a purely data-driven method without biological modeling, in the current study, we have carefully considered the effects of HRF on Granger causality analysis on BOLD-fMRI data. The use of DOI terms in the within-group effective connectivity analysis, the main concern of identifying different effective connectivity patterns between the patients and controls rather than revealing canonical causal structure, as well as the group-level strategy for multisubject Granger causality analysis in the current study, have been suggested by recent analyses that are theoretically useful to relieve the HRF effects (Schippers et al., 2011; Barnett and Seth, 2014). Furthermore, considering that the HRF has been reported to be different in epilepsy subjects (David et al., 2008), we adopted the blind-deconvolution technique proposed by Wu et al. (2013) to deconvolve the mean time courses of the six ROIs (obtained in Section Region of interest definition and time course extraction) for each subject separately, and on the basis of the deconvolved BOLD time courses, we repeated the Granger causality analysis described in Section Evaluating between-group effective connectivity difference. In this case, both the time domain partial Granger causality and the frequency domain PDC have revealed only one effective connectivity that showed significant group difference (Mann-Whitney *U*-tests, *p* < *0.05*, FDR corrected), i.e., the increased causal influence from the rDLPFC to the dACC in the IGE-GTCS patients than controls, which is consistent with our prior result based on the BOLD time courses without deconvolution. We thus infer that, the altered effective connectivity from the rDLPFC to the dACC, which is consistently revealed by the two domains' multivariate Granger causality analyses on both the BOLD and deconvolved BOLD time courses, is probably a key factor associated with cognitive dysfunctions in IGE-GTCS.

### Limitations and future directions

Several limitations in this study should be mentioned. First, due to the absence of neuropsychological tests for both the patients and the controls, we cannot precisely relate the significant Granger causal connectivity to the specific cognitive functions and neuropsychological parameters, the interpretations of the results are simply inferences derived from earlier researches. Second, it is reported that AED toxicity is related to psychopathology and abnormal neuronal function in epilepsy (Schmitz, 1999). In the current study, all patients were treated with AEDs, including 24 patients with monotherapy and 3 patients with polytherapy; the AEDs included sodium valproate (VBA), phenytoin (PHT), carbamazepine (CBZ), lamotrigine (LTG), phenobarbital (PB), and topiramate (TPM). However, we have carefully considered the potential confounding effects of AEDs on ICNs in the study. We ensured that all patients received no medication for at least 48 h prior to the MRI scanning to avoid direct effects of AEDs on the effective connectivity analysis. Nonetheless, the long-term effects of AEDs could not be excluded. Third, a relatively small number of ROIs were used in the current study to investigate the interconnectivity between networks. An extension to a larger set of nodes across different brain networks would be considered in the future. In addition to the above mentioned aspects, future works could also focus on EEG-fMRI multimodal integration for resting-state as well as task-based time-frequency multivariate Granger causality analysis, and evaluate causal relationship between ROIs using dynamic causal modeling (Friston et al., 2003).

## Conclusions

In this study, we conducted combined time and frequency domains multivariate Granger causality analyses to investigate effective connectivity among the key nodes of the three core neurocognitive networks in IGE-GTCS patients and matched healthy controls. The results revealed two SN-involved effective connectivity that exhibited significant group difference. One is the decreased Granger causal influence from the rFIC to the PCC in the patients relative to the healthy controls given by time domain analysis, which may underline impaired cognitive abilities as well as mental inflexibility in IGE-GTCS. Another is the significantly increased Granger causal influence from the rDLPFC to the dACC in patients than controls revealed by both the time and frequency domains analyses. This altered effective connectivity may indicate a disruption to the well-organized cognitive control processes thus probably leading to disorders in working memory, sustained attention, as well as executive dysfunction in IGE-GTCS. The current work proposes a combination framework of time and frequency domains multivariate Granger causality analyses that is suitable for multisubject studies, and demonstrates for the first time that patients with IGE-GTCS exhibited altered Granger causal interactions across the SN, DMN, and CEN, shedding new lights on the psychopathological mechanism of IGE-GTCS.

## Author contributions

DH, SQ designed research; HW, JA, HS, and LZ performed research; HW analyzed the data; and HW, JA, HS, and LZ wrote the paper.

### Conflict of interest statement

The authors declare that the research was conducted in the absence of any commercial or financial relationships that could be construed as a potential conflict of interest.

## Appendix

### Computation of granger causality measures

For better understanding of the calculation process and the definition of each Granger causality measures, we took a general system of *N*(*N* ≥ 3) variables *X*_*i*_(*t*), *i* = 1, 2, …*N* as an example. The unrestricted MVAR model of the system can be written as:

(A3) $$\begin{array}{lll} \left[\begin{array}{l} X_{1}\left(t\right)\\ X_{2}\left(t\right)\\ \vdots \\ X_{N}\left(t\right) \end{array}\right] & = & \overset{p}{\underset{j=1}{\sum }}\left[\begin{array}{cccc} A_{11}\left(j\right) & A_{12}\left(j\right) & \ldots & A_{1N}\left(j\right)\\ A_{21}\left(j\right) & A_{22}\left(j\right) & \ldots & A_{2N}\left(j\right)\\ \vdots & \vdots & \ddots & \vdots \\ A_{N1}\left(j\right) & A_{N2}\left(j\right) & \ldots & A_{NN}\left(j\right) \end{array}\right]\cdot \\ \left[\begin{array}{l} X_{1}\left(t-j\right)\\ X_{2}\left(t-j\right)\\ \vdots \\ X_{N}\left(t-j\right) \end{array}\right]+\left[\begin{array}{l} E_{1}\left(t\right)\\ E_{2}\left(t\right)\\ \vdots \\ E_{N}\left(t\right) \end{array}\right] \end{array}$$

where *p* is the model order, *E*_*i*_, *i* = 1, 2, …*N* are the model residuals (prediction errors), the elements of **A**(*j*) are called regression coefficients. The noise covariance matrix of the unrestricted model can be represented as:

(A4) $$\begin{array}{l} \Sigma =\left[\begin{array}{cccc} \text{var}\left(E_{1}\right) & \text{cov}\left(E_{1},E_{2}\right) & \cdots & \text{cov}\left(E_{1},E_{N}\right)\\ \text{cov}\left(E_{2},E_{1}\right) & \text{var}\left(E_{2}\right) & \cdots & \text{cov}\left(E_{2},E_{N}\right)\\ \vdots & \vdots & \ddots & \vdots \\ \text{cov}\left(E_{N},E_{1}\right) & \text{cov}\left(E_{N},E_{2}\right) & \cdots & \text{var}\left(E_{N}\right) \end{array}\right] \end{array}$$

To measure the Granger causality from *X*_2_(*t*) to *X*_1_(*t*), we delete the row 2 and column 2 of the noise covariance matrix of the unrestricted model, then partition the matrix into blocks:

and omit the time course *X*_2_(*t*) to obtain a restricted MVAR model as:

(A6) $$\begin{array}{lll} \left[\begin{array}{l} X_{1}\left(t\right)\\ X_{3}\left(t\right)\\ \vdots \\ X_{N}\left(t\right) \end{array}\right] & = & \overset{p}{\underset{j=1}{\sum }}\left[\begin{array}{cccc} A_{11}^{{}^{\text{′}}}\left(j\right) & A_{13}^{{}^{\text{′}}}\left(j\right) & \ldots & A_{1N}^{{}^{\text{′}}}\left(j\right)\\ A_{31}^{{}^{\text{′}}}\left(j\right) & A_{33}^{{}^{\text{′}}}\left(j\right) & \ldots & A_{3N}^{{}^{\text{′}}}\left(j\right)\\ \vdots & \vdots & \ddots & \vdots \\ A_{N1}^{{}^{\text{′}}}\left(j\right) & A_{N3}^{{}^{\text{′}}}\left(j\right) & \ldots & A_{NN}^{{}^{\text{′}}}\left(j\right) \end{array}\right]\cdot \\ \left[\begin{array}{l} X_{1}\left(t-j\right)\\ X_{3}\left(t-j\right)\\ \vdots \\ X_{N}\left(t-j\right) \end{array}\right]+\left[\begin{array}{l} E_{1}^{{}^{\text{′}}}\left(t\right)\\ E_{3}^{{}^{\text{′}}}\left(t\right)\\ \vdots \\ E_{N}^{{}^{\text{′}}}\left(t\right) \end{array}\right] \end{array}$$

and the noise covariance matrix of the restricted model is:

Next, we explore the frequency domain representation of MVAR model. The Fourier transform of (A3) gives:

(A8) $$\begin{array}{l} \left[\begin{array}{cccc} A_{11}\left(f\right) & A_{12}\left(f\right) & \ldots & A_{1N}\left(f\right)\\ A_{21}\left(f\right) & A_{22}\left(f\right) & \ldots & A_{2N}\left(f\right)\\ \vdots & \vdots & \ddots & \vdots \\ A_{N1}\left(f\right) & A_{N2}\left(f\right) & \ldots & A_{NN}\left(f\right) \end{array}\right]\left[\begin{array}{l} X_{1}\left(f\right)\\ X_{2}\left(f\right)\\ \vdots \\ X_{N}\left(f\right) \end{array}\right]=\left[\begin{array}{l} E_{1}\left(f\right)\\ E_{2}\left(f\right)\\ \vdots \\ E_{N}\left(f\right) \end{array}\right] \end{array}$$

where the components of the **A***(f)* matrix are:

(A9) $$\begin{array}{l} A_{lm}(f)=\delta_{lm}-\sum_{j=1}^{p}A_{lm}(j)e^{-i2\pi fj}\\ \delta_{lm}=\{\begin{array}{c} 1,\;l=m\\ 0,\;l\neq m \end{array} \end{array}$$

and we rewrite the **A**(*f*) to the following form:

(A10) $$\begin{array}{lll} \text{A}\left(f\right) & = & \left[\begin{array}{cccc} A_{11}\left(f\right) & A_{12}\left(f\right) & \ldots & A_{1N}\left(f\right)\\ A_{21}\left(f\right) & A_{22}\left(f\right) & \ldots & A_{2N}\left(f\right)\\ \vdots & \vdots & \ddots & \vdots \\ A_{N1}\left(f\right) & A_{N2}\left(f\right) & \ldots & A_{NN}\left(f\right) \end{array}\right]\\ =\left[\begin{array}{cccc} {\text{a}}_{1}\left(f\right) & {\text{a}}_{2}\left(f\right) & \ldots & {\text{a}}_{N}\left(f\right) \end{array}\right] \end{array}$$

then the Granger causality measures in two domains can be formalized as follows:

### Time domain partial granger causality

Referring to (A5) and (A7), the partial Granger causality from *X*_2_(*t*) to *X*_1_(*t*), conditioned on all the remaining variables besides *X*_1_(*t*) and *X*_2_(*t*), is given by:

(A11) $$F_{2\to 1|\text{others}}^{P}=\ln\;\frac{\left|\rho_{11}-\rho_{12}\rho_{22}^{-1}\rho_{21}\right|}{\left|\sum_{11}-\;\sum_{12}\sum_{22}^{-1}\sum_{21}\;\right|}$$

which can avoid influence of exogenous inputs and latent variables, and describe the direct causal relationships between time courses (Guo et al., 2008). When there is no direct influence from *X*_2_(*t*) to *X*_1_(*t*), the *A*_12_(*j*), *j* = 1…*p* in (A3) are uniformly equal to zero, leading to $F_{2\to 1|\text{others}}^{P}=0$. On the contrary, when a direct influence from *X*_2_(*t*) to *X*_1_(*t*) exists, we will get $F_{2\to 1|\text{others}}^{P}>0$.

In addition, a difference of influence (DOI) term is introduced to describe the dominant direction of causal influence that measured as the difference (Roebroeck et al., 2005):

(A12) $$\begin{array}{l} D_{2\to 1|\text{others}}^{P}=F_{2\to 1|\text{others}}^{P}-F_{1\to 2|\text{others}}^{P} \end{array}$$

which can further limits potentially spurious links caused by hemodynamic blurring (Seth, 2010).

### Frequency domain partial directed coherence

To measure the Granger causality from *X*_2_(*t*) to *X*_1_(*t*) in frequency domain, the PDC is defined as (Baccalá and Sameshima, 2001):

(A13) $$\begin{array}{l} \pi_{12}\left(f\right)=\frac{A_{12}\left(f\right)}{\sqrt{{\text{a}}_{2}^{H}\left(f\right){\text{a}}_{2}\left(f\right)}} \end{array}$$

It can be seen that the evaluation of Granger causality from *X*_2_(*t*) to *X*_1_(*t*) is discretized into a set of frequency slices. Therefore, by selecting the interested frequency range, we can use PDC to evaluate direct Granger causality between time courses at each discrete frequency.

Additionally, the non-normalized DTF from *X*_2_(*t*) to *X*_1_(*t*) is given by Kaminski et al. (2001):

(A14) $$\begin{array}{l} \theta_{12}^{2}\left(f\right)=\frac{{\left|{\text{M}}_{21}\right|}^{2}}{{\left|\text{A}\left(\text{f}\right)\right|}^{2}} \end{array}$$

where |**M**_**21**_| is a minor of **A(f)** with row 2 and column 1 removed. The DTF in the multivariate condition could not avoid the detection of indirect causal relationship between time courses (Kaminski et al., 2001).
